# Bibliographical Notices

**Published:** 1844-12-14

**Authors:** 


### BIBLIOGRAPHICAL NOTICES.

### REPORTS OF ENGLISH LUNATIC ASYLUMS.

*Thirty-Third Annual Report of the state of the General Lunatic Asylum, near Nottingham, prepared for the Anniversary meeting, Sept.* 1843.

*The Nineteenth and Twentieth Annual Report of the Visitors of the General Lunatic Asylum for the county and city of Gloucester,* 1842 *and* 1843.

*Report of the state of the Kent County Lunatic Asylum, from its opening on the* 1*st of January,* 1833, *to the* 31*st of December,* 1842.

*The Twenty-fourth and Twenty-fifth Reports of the Directors of the West Riding of York Pauper Asylum, Wakefield,* 1843 *and* 1844.

*The Report of the Committee of Visitors of the Lunatic Asylum for the County of Leicester, January,* 1843.

*The Fifteenth Report of the Visiting Justices of the County Lunatic Asylum, at Hanwell.* London, 1841.

*The Sixty-fourth and Sixty-eighth Reports of the Visiting Justices, &c., &c.* London, 1842 & 1843.

*Report of the Lunatic Asylum for the County of Lancaster for* 1841, 1842 *and* 1843.

*Observations on the necessity of an extended Legislative protection of persons of unsound mind.* By Edward D. De Vitré, M. D., Physician to the Lancaster Lunatic Asylum. London, 1843.

*Announcement of the Association of Medical Officers of Hospitals for the Insane, with Recommendations for filling up the Register of cases agreed to at the Annual Meeting, held at the Asylum, Lancaster.* June 2d and June 3d, 1842.

*Report of the Metropolitan Commissioners in Lunacy to the Lord Chancellor. Presented to both Houses of Parliament by command of Her Majesty,* 8vo. p. 291. London, 1844.

We have been favored with the valuable documents in relation to the Insane, of which the above are the titles, by Professor Dunglison. They were sent to him by a zealous and intelligent young philanthropist of Boston, Mr. Samuel M. Barnard, who, from no other feelings than those of benefiting his fellow man afflicted with the most grievous of all calamities, has established, at much labour and expense, a system of exchanges amongst those who are entrusted with the care of establishments for the insane on both sides of the Atlantic.

These admirable reports show, in bold relief, the decided improvement in the mode of managing those unfortunate subjects for our commiseration; and, with the Reports of our own excellent Institutions of a like nature, sufficiently exhibit the gratifying fact, that insanity, when taken early, is in the large majority of cases a highly curable disease; but, when improperly or too tardily treated, inflicts perpetual misery on the sufferer, and compels him, for the remainder of his existence, to be deprived of al intercourse with his sane brethren.

As Pennsylvanians, it pains us, when we look at our destitute condition at home, to observe so many excellent Institutions for the insane poor, not only in different states of this Union, but especially in Great Britain. When shall we be able to carry into effect that wise act of the Legislature, which sanctioned the building of such an institution, but nipped it in the very bud by subsequent—we do not say improper or uncalled for—interference? Every year delayed is a sad loss to humanity; renders maladies intractable, which under better circumstances might have been removed without difficulty; and subjects to perpetual confinement those who might have been valued members of society.

It is pleasing to see the spirit of philanthropy breathed through these reports;—the elevation given to man, even insane man, by the judicious moral treatment every where now recommended; the abandonment of almost all forms of coercion, and of every kind of irrational severity, so barbarously put in force until within recent periods. For much of this amelioration, the world is indebted to the amiable and accomplished medical superintendent of the Hanwell Lunatic Asylum,—Dr. Conolly.

Our limits would not permit us, did we desire it, to notice the contents of all the *Brochures* before us; but we shall indulge in a few remarks in regard to one or two of them.

A great difficulty has hitherto existed, in forming any accurate comparison of results, in consequence of a different registration of cases being adopted by recorders. The following classes are recommended by the "Association of Medical Officers of Hospitals for the Insane."

Class 1. Cases of the first attack, of not more than three months' duration.

Class 2. Cases of the first attack, of more than three, but of not more than twelve months' duration.

Class 3. Cases not of the first attack, and of no more than twelve months' duration.

Class 4. Cases whether of the first attack or not, of more than twelve months' duration.

As regards the form of mental disorder, it is recommended, as much as may be, to refer every case to one of the following primary forms. 1. Mania. 2. Melancholia. 3. Manomania. 4. Moral insanity. 5. Dementia, under the two heads of Imbecility and Fatuity. 6. Congenital Idiocy.

*Criminal Lunatics* should be farther distinguished as such. On the whole, perhaps, this is as good a system of registration as can be adopted.

The observations of Dr. Vitré contain much interesting matter. From them we learn, that the parliamentary returns of pauper lunatics for England and Wales, made in the year 1837, show that there were then, in all, 13,667 paupers of unsound minds, chargeable to their respective parishes. Of these, 4,271 were confined in public and private asylums, whilst the remaining 9,396 were under the care and management of the guardians of the poor, and were maintained as in-door and out-door paupers,"—being about one pauper lunatic to every thousand of the whole population, according to the census of 1831; and we think it highly probable, that the ratio is not much short of this in Pennsylvania.

The "Report of the Metropolitan Commissioners in Lunacy to the Lord Chancellor," is really a most valuable document. It affords a sketch of the different classes of Lunatic Asylums, their construction, condition, management and visitation;—of the County Asylums, partly supported by contributions;—of naval and military hospitals;—of public hospitals supported wholly or in part by voluntary contributions;—of licensed houses;—of abuses and defects;—condition of paupers on admission; forms of disease; medical treatment; diet; classification of lunatics; occupations, amusements, and exercises; restraints; religious services; on the admission and liberation of patients; statistics of insanity; criminal lunatics; suggestions for the amendment of the law, &c., &c.

We extract the general statement of insane persons confined in asylums of England and Wales, Jan. 1, 1844.

| | | *Males.* | *Females.* | *Total.* |
|---|---|---|---|---|
| Private patients, | | 1989 | 1801 | 3790 |
| Paupers, | | 3532 | 3950 | 7482 |
| Total, | | 5521 | 5751 | 11272 |
| State as to probability of recovery. Curable. | Private, | 492 | 553 | 1045 |
| | Pauper, | 687 | 787 | 1474 |
| | Total, | 1179 | 1340 | 2519 |
| Incurable. | Private, | 1497 | 1248 | 2745 |
| | Pauper, | 2834 | 3157 | 5991 |
| | Total, | 4331 | 4405 | 8736 |
| Epileptics, | | 575 | 376 | 951 |
| Idiots, | | 347 | 251 | 598 |
| Homicidal Patients, | | 180 | 98 | 278 |
| Suicidal Patients, | | 303 | 393 | 696 |
| Civil state. | Married, | 1501 | 1664 | 3165 |
| | Single, | 3346 | 2982 | 6328 |
| | Widowed, | 340 | 798 | 1138 |
| | Not known, | 212 | 197 | 409 |
| Class of life, and previous occupation. | Upper & middle classes, | 1389 | 1315 | 2704 |
| | Agricultural, | 1183 | 469 | 1652 |
| | Artisan and In-door, | 1640 | 2228 | 3868 |
| | Others, | 1187 | 1629 | 2816 |
| Criminal Lunatics, | | 202 | 55 | 257 |
| Found Lunatic by Inquisition, | | 146 | 87 | 233 |

The appendix contains an account of the accommodation, and cost of erection of County Asylums;—the condition of pauper patients on admission into the asylums;—the dietaries of such patients, &c. &c. &c.

---

*Lecture introductory to the course of Medical Chemistry in the Medical Department of Pennsylvania College, Philadelphia. For the Session* 1844-5. By Washington L. Atlee, M. D.

This is a spirited and well written production, in which the author contends with becoming zeal for the importance of chemistry, in its relations to the science and practice of Medicine. He very properly maintains that the instruction given to medical students in this branch, should be such as will qualify them for the discharge of the duties of a physician, rather than that required in the various mechanical arts. He observes—"It is time, I think, that Medical Chemistry should be taught in *medical* schools, as it is entitled to paramount importance to the *medical* student." Surely our author does not mean to say that *medical* chemistry has not been taught by any one heretofore! This sentence, in connexion with the peculiar phraseology of the title of the lecture, seems to imply that opinion—if so, we must frankly say that he is mistaken. It is a great improvement in the organization of Medical Colleges that the chemical chair is now very generally occupied by *physicians*—gentlemen frequently of great experience as practitioners, and therefore fully acquainted with the wants of the student of medicine—so that Professor Atlee has *example* as well as countenance for the course he proposes to pursue in teaching his branch.

The views expressed in this lecture generally are such as as we can approve. On one point, however, we dissent—viz., "There is a well grounded supposition," the author remarks, "that the injurious effects of malarious districts are dependent upon the presence of this compound gas" (Sulph. Hydrogen.) This suggestion, we are aware, was made a few years ago by a distinguished European, but we had thought it was every where rejected as inconsistent with undoubted facts. If the hypothesis were true, in what a deplorable condition would be the inhabitants of large cities, who are continually breathing an atmosphere highly charged with this offensive gas—and yet such places are the most exempt, cæteris paribus, from malarious diseases.

It is evident from various passages in this address, that Professor Atlee subscribes to the peculiar chemico-physiological doctrines of Liebig, more especially in reference to the source of animal heat. On this point he certainly is on the fashionable side of the question, and probably the true one, although there are some sturdy opponents. We regret that our limits forbid a more extended notice of this very creditable production—in matter and manner, it is in strong contrast with the egotism, spleen, and twattling, that characterize some other addresses of the kind, which, unfortunately for their authors, have recently been published.

---

*The Physiology of the London Medical Student, and Curiosities of Medical Experience.* By "Punch." With illustrations by Leech. Philadelphia: Carey & Hart.

This is a small volume of ninety six pages, written and illustrated in the true Punch style. It is evidently designed, like the other productions of the same popular school, for general reading, although Medical Students, their pursuits, pastimes, tricks and irregularities, are the subjects. As might be expected, every thing is exaggerated, coloured, turned, twisted, and made to assume a comical and ridiculous form; nevertheless there are some very good hints,—some useful lessons,—which young men of every class and pursuit might study with advantage.

